# Tolerability of a piezoelectric microneedle electroporator in human subjects

**DOI:** 10.1002/btm2.10662

**Published:** 2024-03-14

**Authors:** Chao‐Yi Lu, Pankaj Rohilla, Eric I. Felner, Gaurav Byagathvalli, Erkan Azizoglu, M. Saad Bhamla, Mark R. Prausnitz

**Affiliations:** ^1^ Wallace H. Coulter Department of Biomedical Engineering at Georgia Tech and Emory University, Georgia Institute of Technology Atlanta Georgia USA; ^2^ School of Chemical and Biomolecular Engineering, Georgia Institute of Technology Atlanta Georgia USA; ^3^ Department of Pediatrics, Division of Endocrinology Emory University School of Medicine Atlanta Georgia USA

**Keywords:** DNA/RNA vaccination, electroporation, human skin tolerability, microneedle electrode array

## Abstract

Electroporation, or the use of electric pulses to facilitate the intracellular delivery of DNA, RNA, and other molecules, is a well‐established technique, that has been demonstrated to significantly augment the immunogenicity of DNA/mRNA vaccines and therapeutics. However, the clinical translation of traditional electroporators has been limited due to high costs, large size, complex user operation, and poor tolerability in humans due to nerve stimulation. In prior work, we introduced ePatch: an ultra‐low‐cost, handheld, battery‐free electroporator employing a piezoelectric pulser coupled with a microneedle electrode array that showed enhanced immunogenic responses to an intradermal SARS‐CoV‐2 DNA vaccine in mice. The current study shifts focus from efficacy to tolerability, hypothesizing that ePatch's microneedle array, which localizes the electric field to the superficial skin strata, will minimize nerve stimulation and improve patient comfort. We tested this hypothesis in 14 healthy adults, monitoring pain and other potential adverse effects associated with electroporation. Compared to the insertion of a traditional hypodermic needle, the ePatch was less painful. Adverse effects such as pain, tenderness, erythema and swelling at the application sites were minimal, transient, and statistically indistinguishable between the experimental and placebo ePatch application, suggesting excellent tolerability towards electroporation. In summary, ePatch has a favorable tolerability profile in humans and offers the potential for the safe use of electroporation in a variety of clinical settings, including DNA and mRNA vaccination.

**Translational Impact Statement**

In this article, our findings suggest excellent tolerance of electroporation with an ultra‐low cost electroporator (ePatch) in human subjects. Building on previous research involving a DNA‐based SARS‐CoV‐2 vaccine delivery in rodents, where electroporation via the ePatch elicited robust antibody responses at a 10‐fold dose sparing compared to intramuscular administration without electroporation, our current findings represent a pivotal advancement in translating this technology for the delivery of nucleic‐acid‐based vaccines in humans.

## 1 INTRODUCTION

The development and use of nucleic acid‐based vaccines and therapeutics has increased rapidly, underscored by the widespread deployment of mRNA‐ and DNA‐based vaccines against SARS‐CoV‐2.^1^, ^2^ Unlike traditional subunit or attenuated live virus vaccines, these nucleic acid‐based vaccines can be developed in much less time, which is essential during sudden pandemics such as the recent outbreak of coronavirus disease‐19 (COVID‐19). Notably, the Moderna and Pfizer/BioNTech mRNA vaccines were designed and authorized for human use in less than a year^3^ as were adenoviral vector DNA vaccines like those by Janssen/Johnson & Johnson and Oxford/AstraZeneca.^4^

Advancements extend beyond vaccines to DNA gene therapies and RNA treatments. With the commercial debut of Gendicine for cancer in 2003,^5^ the approval of gene therapies has accelerated, with significant milestones including Glybera's approval in Europe in 2012 for lipoprotein lipase deficiency,^6^ Luxturna's US approval in 2017 for a genetic retinal disease,^7^ and Zolgensma's approval in the United States and Japan in 2019 for spinal muscular atrophy.^8^ In addition to these approved gene therapy drugs, gene delivery is also performed ex vivo for clinical cancer immunotherapies (e.g., CAR‐T cell treatment) such as Kymriah and Yescarta.^9^

Concurrently, RNA gene therapies have advanced rapidly, with antisense oligonucleotides (ASOs), small interfering RNAs, and RNA aptamers,^10^, ^11^ leading to the approval of drugs like Leqvio in the United States in 2021.^12^ More RNA‐based medications are likely to be commercially available in the near future, given the increase in the number of RNA drugs in development and being evaluated in clinical trials.^11^, ^13^

However, the intracellular delivery of genetic material remains a challenge. While current DNA vaccines predominantly use viral vectors for their high transduction efficiency,^14^, ^15^ interest in non‐viral vectors persists due to advantages in safety, cost, and capacity.^13^, ^15^ The approved mRNA vaccines for SARS‐CoV‐2, for instance, employ lipid nanoparticles (LNPs), which are a non‐viral vector delivery system. Although LNPs can offer good scalability, high transfection efficiency, and biocompatibility, they are susceptible to instability in vivo and rapid clearance from the body.^13^ The commercialization of LNPs has been further constrained by costly and complex manufacturing, the need for frozen storage and distribution, and the difficulties in determining appropriate LNP formulations and RNA modifications.^16^ Most failures in clinical translation of LNP‐encapsulated RNA are due to the instability of these systems under varying conditions (e.g., inside the endosome), the lack of targeting ability that can lead to off‐target side effects and lower therapeutic efficacy, and/or the inability of efficient endosomal escape.^16^

Electroporation is an attractive alternative to improve the delivery of DNA and RNA to cells, circumventing the limitations of viral vectors and LNPs by directly introducing nucleic acids into cells through transient membrane pores.^17^, ^18^, ^19^, ^20^ This can overcome certain safety, efficacy, and cargo size limitations associated with DNA delivery via viral vectors. In addition, electroporation does not require optimization of LNP formulations for the delivery of a specific RNA strand, enabling a payload‐agnostic approach. Rather than relying on a vector for gene delivery, electroporation employs electric pulses that temporarily disrupt cell membranes to facilitate molecular transfer into cells. For more than four decades, this method has been widely employed in laboratories as a standard in vitro transfection technique to introduce genetic material into cells.^21^ In addition to DNA delivery, electroporation can also be used to deliver mRNA, proteins, molecular probes, or nanodevices intracellularly.^22^, ^23^

Electroporation is currently approved in Europe for electrochemotherapy.^24^ The first human application of electroporation involved the intracellular delivery of bleomycin, a cytotoxic drug used to treat dermatologic tumors, that is unable to pass through intact cell membranes.^25^ More recently, researchers have been interested in other uses of electrochemotherapy, such as the treatment of deeper tumors during an open surgery procedure.^25^, ^26^ Electrogene transfer is yet another potential use of electroporation in the clinical setting.^26^ In early 2023, more than 20 clinical trials were approved for investigating electrogene transfer of plasmid DNA to treat cancer or vaccinate against diseases.^17^, ^26^, ^27^ In addition to plasmid DNA delivery, preclinical studies have also been conducted on the use of electroporation for RNA delivery, specifically for vaccination^28^, ^29^ and cancer treatment.^30^, ^31^

Despite its promise, the clinical application of electroporation is often constrained by the complexity and cost of the technology. Electroporators for clinical use are complex, expensive (i.e., cost thousands of dollars), require a power supply, and are not easily portable (weighing >5 kg).^32^ The high cost and complexity of using electroporation clinically pose a barrier to accessing this technology for patients, providers, and payers, especially in low‐resource settings. Furthermore, electroporation has been reported to cause pain and/or muscle contractions, which can reduce patient acceptance and compliance with electroporation.^20^, ^33^, ^34^, ^35^ To address these concerns, we have developed an ultra‐low‐cost and portable electroporator called ePatch.^32^

ePatch is an inexpensive (<$1) and light (<50 g), handheld device.^32^ It consists of two major components: a piezoelectric pulser and a microelectrode array (MEA). The electric pulser used in this study utilizes an inexpensive piezoelectric crystal to generate a high‐voltage pulse, as previously described.^32^, ^36^ The MEA consists of a 6‐by‐9 array of microneedles measuring 650 μm in length and is designed to target delivery to antigen‐presenting cells (APCs) and other cells in the viable epidermis layer just below the skin surface. The estimated cost of microneedle electrodes per unit is less than $0.10 when produced in bulk, for example using lithographic etching technology on stainless steel sheets.

Due to the length of the microneedles, the electric field is limited to the epidermis and superficial dermis, thus avoiding stimulation of the sensory and motor nerves located deeper in the dermis or muscle tissue. The nominal electric field strength in the tissue targeted by ePatch is ~2000–3000 kV/cm,^32^ similar to the electric field strength used for microsecond‐long electroporation pulses.^37^, ^38^, ^39^ In a recent study, we showed that electroporation by ePatch elicited at least a 10‐fold dose‐sparing antibody response against SARS‐CoV‐2 compared to intramuscular or intradermal injections of a SARS‐CoV‐2 DNA vaccine in mice.^32^

This study extends our investigation into the tolerability of ePatch in humans compared to hypodermic needle control. We hypothesize that the ePatch will be well‐tolerated in humans, causing little or no nerve stimulation due to the shallow insertion depth of the microneedle electrodes. In this study, subjects rated the pain experienced during ePatch administration using a visual analog scale (VAS) pain score. We also assessed pain, tenderness, erythema, and swelling at the administration site immediately and 24 h after electroporation.

## 2 MATERIALS AND METHODS

### 2.1 Fabrication of ePatch

**FIGURE 1:**
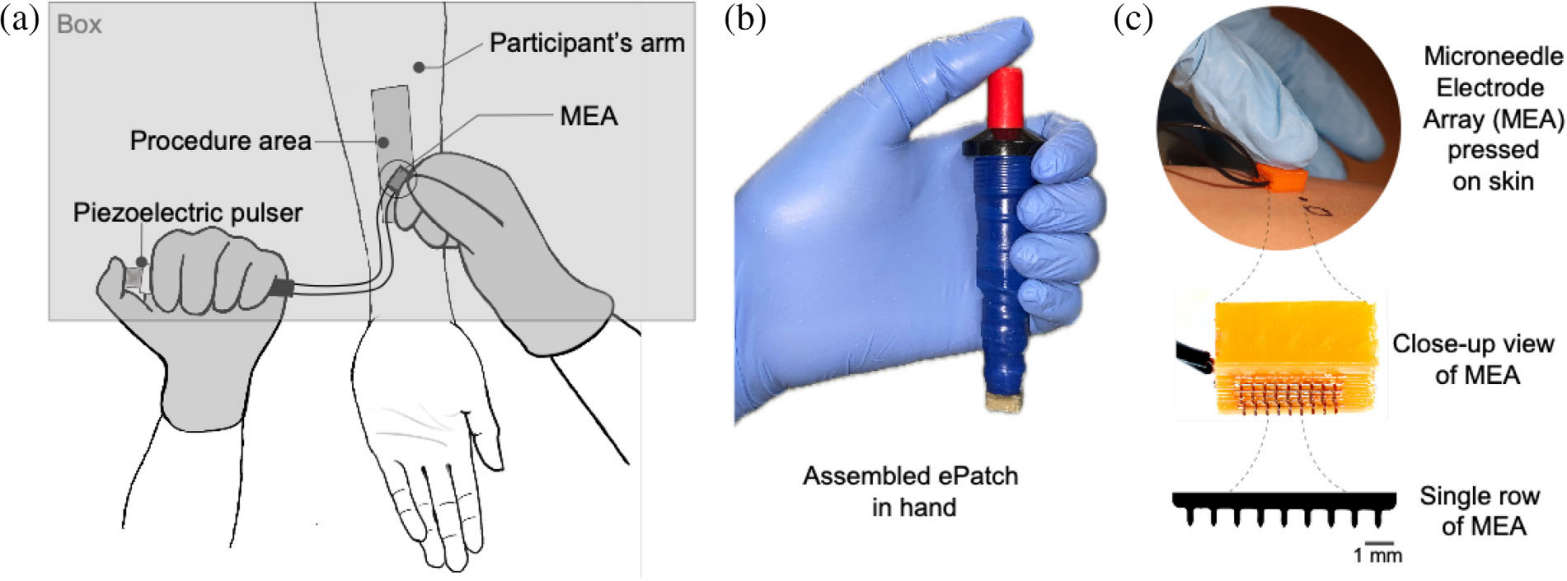
ePatch administration to human subjects. (a) Schematic showing the experimental setup used to perform ePatch application to human subjects. (b) Assembled ePatch held in investigator's hand for a size comparison. (c) Representative photographic images of: the MEA of an ePatch applied on the skin of a study participant, an MEA with exposed microneedles, and a single row of 9 stainless steel microneedles.

ePatches were assembled by connecting a piezoelectric pulser to a MEA, both of which were prepared separately. The MEA was constructed by placing 6 rows of 9 stainless steel microneedles (Tech Etch, Plymouth, MA) in a parallel formation with a consistent gap of 0.9 mm between microneedle rows inside a polylactic acid (PLA) holder, which was 3D printed with an Ultimaker‐3 3D printer (Ultimaker, Geldermalsen, Netherlands). The length of the microneedles was 650 μm and microneedles within the same row had a spacing of 0.8 mm. The MEAs were then connected through two wires to form three pairs of electrodes in parallel rows. The assembled MEAs were placed in sterilization pouches prior to a 24‐h ethylene‐oxide sterilization cycle. The electric pulses were generated via a pulser utilizing an inexpensive piezoelectric crystal as described previously in an earlier study.^26^, ^32^ Before each study, a piezoelectric pulser was connected to an MEA to fully assemble the ePatch (Figure 1).

### 2.2 Human study approval and study subjects

This study was approved by the Institutional Review Board (IRB) of the Georgia Institute of Technology. Informed consent was obtained from all subjects prior to participation in the study. To be included in the study, participants were healthy, nonpregnant adults without a medical condition or taking a medication that could affect pain perception or involved abnormal skin at the treatment sites.

### 2.3 Human study experimental design and procedure

At the beginning of the study, five sites were identified and marked on the forearm of each subject. A box was used to obscure the subject's view of the procedure to ensure blinding. Subjects were asked to put their forearm through a hole in the box when the procedures were conducted (see Figure 1a). The clinical investigator applied the ePatch on the forearm of the human subjects with an estimated force of ~12 N (see Figure S2, Supplementary Information). Immediately after administration, participants verbally reported pain using a visual analog scale (VAS) with a score ranging from 0 (no pain) to 10 (worst imaginable pain).^40^ VAS scores were then normalized with respect to the pain experienced during the administration of hypodermic needle (VAS_HN_) and during the administration of ePatch without any pulses (VAS_NP_). A photograph of the skin was taken immediately and 10 min after administration using a digital camera (Canon Rebel T7i with an 18–55 mm lens). The investigators then visually inspected the skin to assess the size and intensity of erythema and intensity of induration/swelling after administration based on a skin score sheet (see Table S1, Supplementary Information), while residual pain and tenderness were evaluated from the response of the participant. Note that the pain mentioned in the skin scoring system refers to the pain at the site of the procedure after the procedure's completion, not the pain reported during ePatch application.

A digital oscilloscope (SDS 1202X‐E, SIGLENT, Shenzhen, China) measured and recorded the voltage profiles of the electric pulses applied to the skin during procedures involving ePatch at a sampling rate of 1 GSa/s. The electric pulses delivered by ePatch to the skin of human participants showed an oscillating profile with an initial peak voltage of ∼180 V over the course of an initial positive phase of the voltage profile having a duration of ~10 μs (see Figure S2, Supplementary Information). This was followed by a negative‐phase oscillation of similar duration and a peak negative voltage of approximately ~190 V.

In this single‐blind study, two experimental procedures and three control procedures were administered in each subject. The two experimental procedures involved skin electroporation with an ePatch administering 1 pulse and 10 pulses. The three control procedures included

1. a 25‐gauge, 1.6 cm long hypodermic needle fully inserted up to its hub into the skin,
2. insertion of an MEA without an ePatch and
3. insertion of an MEA with 10 pulses administered by a disconnected ePatch (i.e., producing the clicking sound of an ePatch, but without delivering pulses to the subject).

The sequence of administration of these five procedures was randomized for each subject. After 24 h, nine of the participants returned to have their skin assessed using the skin scoring system and to take photographs of the skin study sites (see Table S1, Supplementary Information).

### 2.4 Electroporation of rat skin

All animal experiments were conducted with approval of the Institutional Animal Care and Use Committee (IACUC) of the Georgia Institute of Technology. Adult female Wistar rats (250–300 g, Charles River Laboratories, Wilmington, MA) were kept in a 12 h/12 h light/dark cycle at the animal care facility, given free access to food and water, and acclimatized for at least 7 days before the experiments.

For dorsal hair removal, rats were anesthesized with 5% isoflurane in O_2_ by isoflurane vaporizer (Surgivet Model 100, Smiths Medical, Dublin, OH) supplied through a standard rodent mask. During anesthesia, 1 to 2% isoflurane was supplied, during which dorsal dermal hair of rats was shaved, followed by the application of depilatory cream (Nair, Church & Dwight, Ewing, NJ) for 3 min. The depilatory cream was removed with a wet gauze to clean the skin. One day after hair removal, the dorsal skin of the rats was electroporated using an ePatch of the same design as used in human studies with 10 electric pulses administered in the same way as in human studies. The skin was imaged photographically (Canon Rebel T7i) immediately after electroporation, with follow‐up imaging for up to three more consecutive days.

The rats were euthanized after 4 days, and skin was harvested using an 8 mm biopsy punch. The tissue was fixed in 10% formalin, stained with hematoxylin/eosin (H&E) and imaged for histological analysis (Histowiz, Brooklyn, NY).

### 2.5 Statistical methods

Statistical analysis was performed in R using RStudio Version 2023.06.1 + 524 (RStudio, PBC, Boston, MA). The Shapiro–Wilk test was performed to check the normality of the VAS scores within different study groups. The Wilcoxon rank sum test (two samples, unpaired) was used to compare study groups of different procedures in the form of two independent study groups.^41^ Statistical significance was established with *p*‐values less than 0.05.

## 3 RESULTS

### 3.1 Application of ePatch to human subjects

The ePatch was applied to the forearm of 14 healthy adults (8 F/6 M; age 21–31 year) as shown in Table S2 (Supplementary Information). No unsolicited or serious adverse events were observed in the study.

### 3.2 Pain associated with ePatch reported by human subjects

**FIGURE 2:**
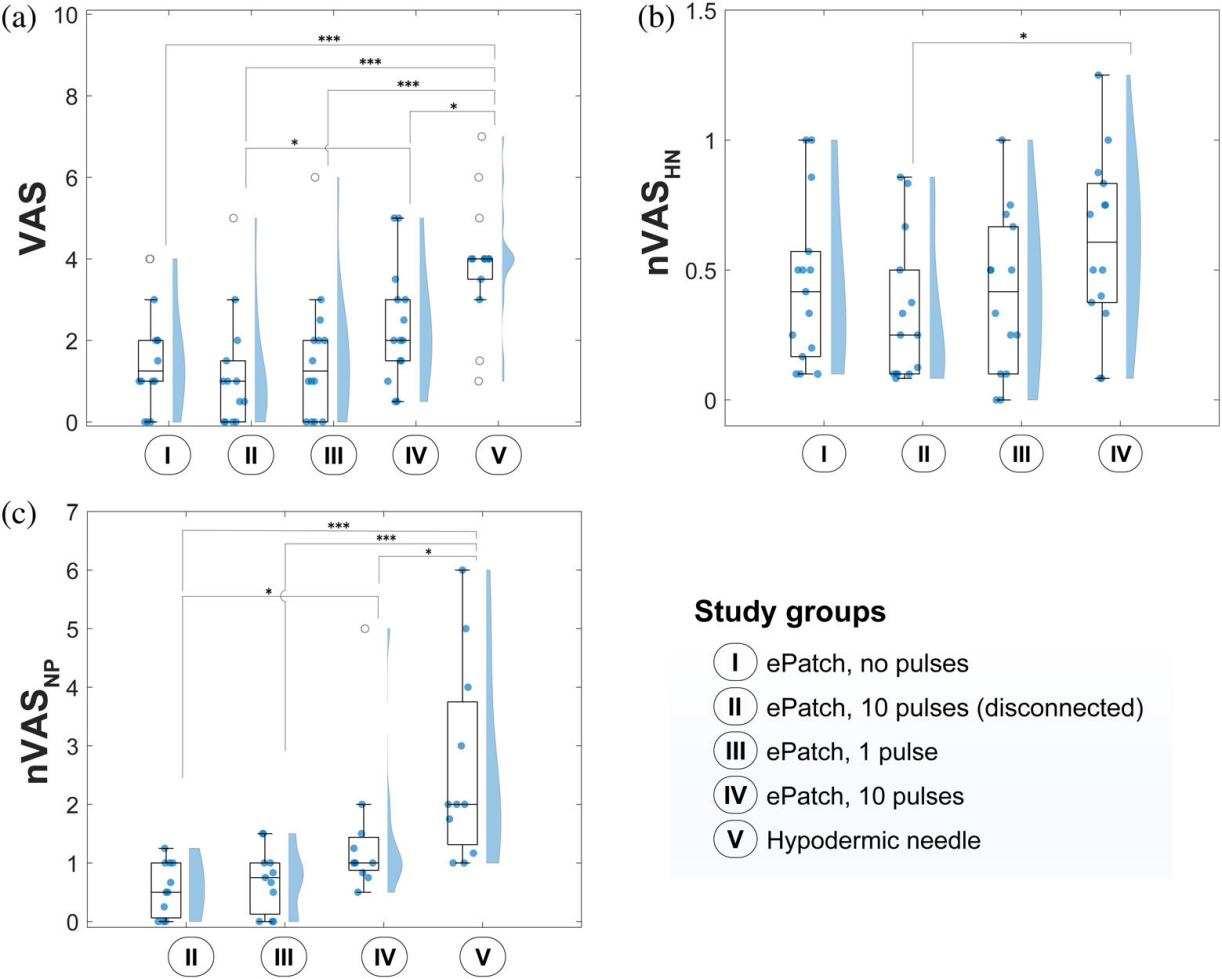
Visual analog scale (VAS) pain scores of ePatch compared with hypodermic needle control in human subjects. VAS scores reported by study participants for five different study groups: (a) absolute VAS scores (*n* = 14), (b) VAS scores normalized to VAS scores experienced during insertion of a 25G hypodermic needle control (nVAS_HN_) (*n* = 14) and (c) VAS scores normalized by VAS scores experienced by application of an ePatch without pulses (nVAS_NP_) (*n* = 11 since the VAS for ePatch with no pulses was zero for three participants). The score of each subject was normalized to their own baseline score. In addition to box and whisker plots, and semi‐violin plots, individual data points (represented by filled circles) are also presented. The box indicates the range between the 25th and 75th percentiles, while the line inside the box represents the median. The whiskers show the maximum and minimum values of the dataset. The outliers are denoted by empty circles, which indicate values that exceed 1.5 times the interquartile range (the range between the 25th and 75th percentiles). In addition to box plots, semi‐violin plots represent the density of the data where the width indicates the frequency of the data points. Statistical significance was determined by the Wilcoxon rank sum test: *p < 0.05, **p < 0.01, and ***p < 0.001.

After the ePatch was applied to human subjects, we collected VAS pain scores to quantify any pain experienced by the participants, ranging from 0 representing “no pain” to 10 representing “the worst imaginable pain.” The VAS score for hypodermic needle insertion into skin (3.93 ± 1.53) was significantly higher than reported for ePatch application with 1 pulse (1.57 ± 1.62, *p* = 0.0005) and with 10 pulses (2.36 ± 1.43, *p* < 0.001) (Figure 2a). The hypodermic needle insertion also had a significantly higher VAS score compared to the negative‐control groups of ePatch application without pulses (1.61 ± 1.33, *p* < 0.001) and ePatch application with 10 disconnected pulses (1.18 ± 1.40, *p <* 0.001). VAS scores for the ePatch application with 1 or 10 pulses showed no significant difference from the two scores for the two negative control ePatch groups.

The data indicate that the study participants found ePatch to be significantly less painful compared to a hypodermic needle and that they did not experience more pain from an ePatch actively administering pulses compared to a placebo ePatch administering no pulses. These findings support the hypothesis that ePatch causes little or no pain in human subjects. It also indicates that any pain reported by subjects was mostly due to the application of the ePatch MEA into the skin and not due to the electroporation pulses.

To better understand how study participants rated the pain of ePatch to that of the hypodermic needle, we normalized ePatch VAS scores for each participant by their VAS score for hypodermic needle insertion, that is, nVAS_HN_ (Figure 2b). This normalized VAS score had a value less than one for every study participant for every ePatch treatment with no pulses, 1 pulse, or 10 disconnected pulses, and for 12 out of 14 subjects (86%) for ePatch treatment with 10 pulses, further indicating that the study participants generally found ePatch to be less painful than a hypodermic needle. On average, nVAS_HN_ values were 0.44 ± 0.32, 0.33 ± 0.28, 0.40 ± 0.31, and 0.60 ± 0.34 for ePatch with no pulse, 10 disconnected pulses, 1 pulse, and 10 pulses, respectively. Within the four normalized groups, there were no significant differences (*p* > 0.05) other than ePatch with 10 disconnected pulses, which had a significantly smaller nVAS_HN_ value than ePatch with 10 pulses (*p* < 0.05).

We also further examined how study participants rated the pain of the various treatments to that of the most benign treatment and therefore normalized VAS scores for each participant by their VAS score for the ePatch with no pulses, that is, nVAS_NP_ (Figure 2c). Most study participants gave an nVAS_NP_ score (i) less than one for ePatch with 1 pulse or 10 disconnected pulses, (ii) close to or slightly above one for ePatch with 10 pulses and (iii) much greater than one for the hypodermic needle. The average nVAS_NP_ scores were 0.56 ± 0.46, 0.70 ± 0.55, and 1.44 ± 1.25, for ePatch with 10 disconnected pulses, 1 pulse and 10 pulses, respectively, while the average score for hypodermic needle insertion was 2.63 ± 1.68. Statistical comparisons showed that nVAS_NP_ for hypodermic needle insertion was significantly greater than for ePatch administration with 10 disconnected pulses (*p* < 0.001), with 1 electric pulse (*p* < 0.001), and ePatch with 10 pulses (*p* < 0.05). Moreover, nVAS_NP_ for ePatch with 10 pulses was significantly greater than ePatch with 10 disconnected pulses (*p* < 0.05).

### 3.3 Tolerability of ePatch in human subjects

We further evaluated the tolerability of ePatch application to the skin 10 min after and 1 day after skin treatment by assessing (i) residual pain, (ii) tenderness, (iii) size and intensity of erythema, and (iv) induration/swelling at the sites of ePatch and hypodermic needle application to the skin.

**FIGURE 3:**
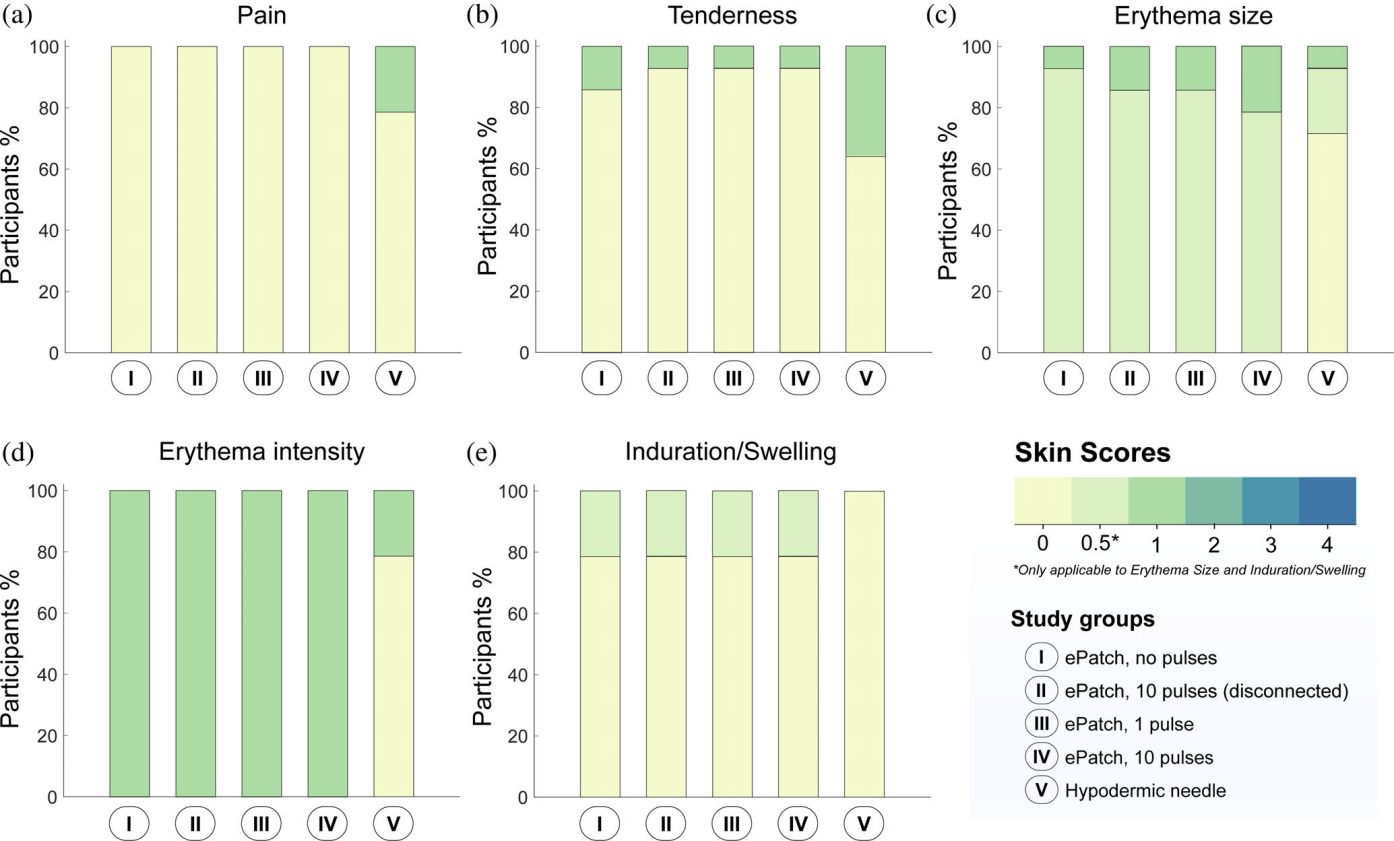
Skin tolerability immediately after application of ePatch and hypodermic needle control in human subjects. Ten minutes after skin treatment, (a) pain and (b) tenderness were assessed subjectively by study participants and (c) erythema size, (d) erythema intensity, and (e) induration/swelling were assessed visually by the clinical investigator and evaluated using a 0–4 point scale score sheet (see Table S1, Supplementary Information) (*n* = 14).

**FIGURE 4:**
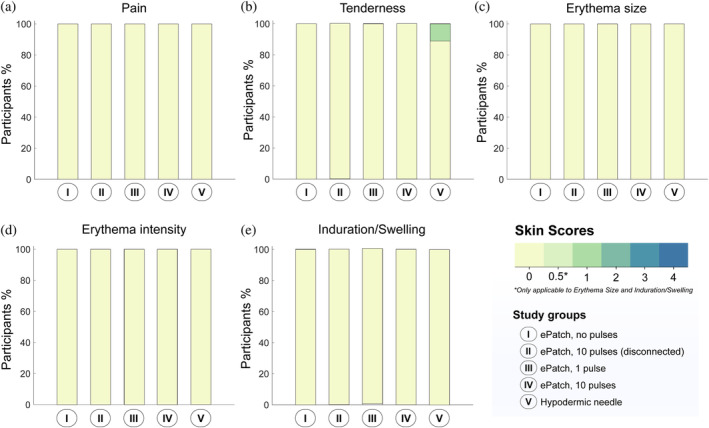
Skin tolerability 1 day after application of ePatch and hypodermic needle control in human subjects. One day after skin treatment, (a) pain, and (b) tenderness were assessed subjectively by study participants and (c) erythema size, (d) erythema intensity, and (e) induration/swelling were assessed visually by a study investigator and evaluated using a 0–4 point scale score sheet (see Table S1, Supplementary Information) (*n* = 9).

*Pain*: In addition to the pain scores reported during ePatch administration in Figure 2, residual pain was also assessed 10 min after skin treatment and presented in Figure 3a. None of the participants experienced residual pain after any of the four ePatch procedures; however, 3 of 14 participants (21%) reported mild residual pain (i.e., VAS score of 1) after insertion of the hypodermic needle. When assessed 1 day after the treatment, no residual pain was reported by any participant (Figure 4a).

*Tenderness*: When skin tenderness was evaluated, either 1 (7%) or 2 (14%) study participants reported mild tenderness (score of 1) 10 min after each ePatch treatment (Figure 3b) and no participants reported tenderness the next day (Figure 4b). In contrast, 5 participants (36%) reported mild tenderness 10 min after hypodermic needle insertion (Figure 3b) and one subject (7%) reported tenderness after 1 day (Figure 4b).

*Erythema size and intensity*: All participants who received the ePatch exhibited mild erythema (score of 1), with the majority (11 to 13 out of 14; 79%–93%) displaying erythema over an area less than 1 cm across (score of 0.5) (Figure 3c,d). For the hypodermic needle control, 10 of 14 participants (71%) had no erythema (score of 0), while 3 participants (21%) had mild erythema (score of 1) over an area less than 1 cm across (score of 0.5) and 1 participant (7%) had mild erythema (score of 1) over an area less than 1.5 cm across (score of 1) (Figure 3c,d). No erythema was detected 1 day after any of the treatments (see Figure 4c,d).

*Induration/swelling*: Skin examined for induration and swelling (i.e., associated with inflammation) yielded 3 participants (21%) in each of the ePatch groups with mild induration/swelling (score of 0.5), while no induration/swelling was observed in the hypodermic needle control sites (Figure 3e). The next day, no induration/swelling was seen in any of the participants (Figure 4e).

### 3.4 Skin imaging in human subjects

**FIGURE 5:**
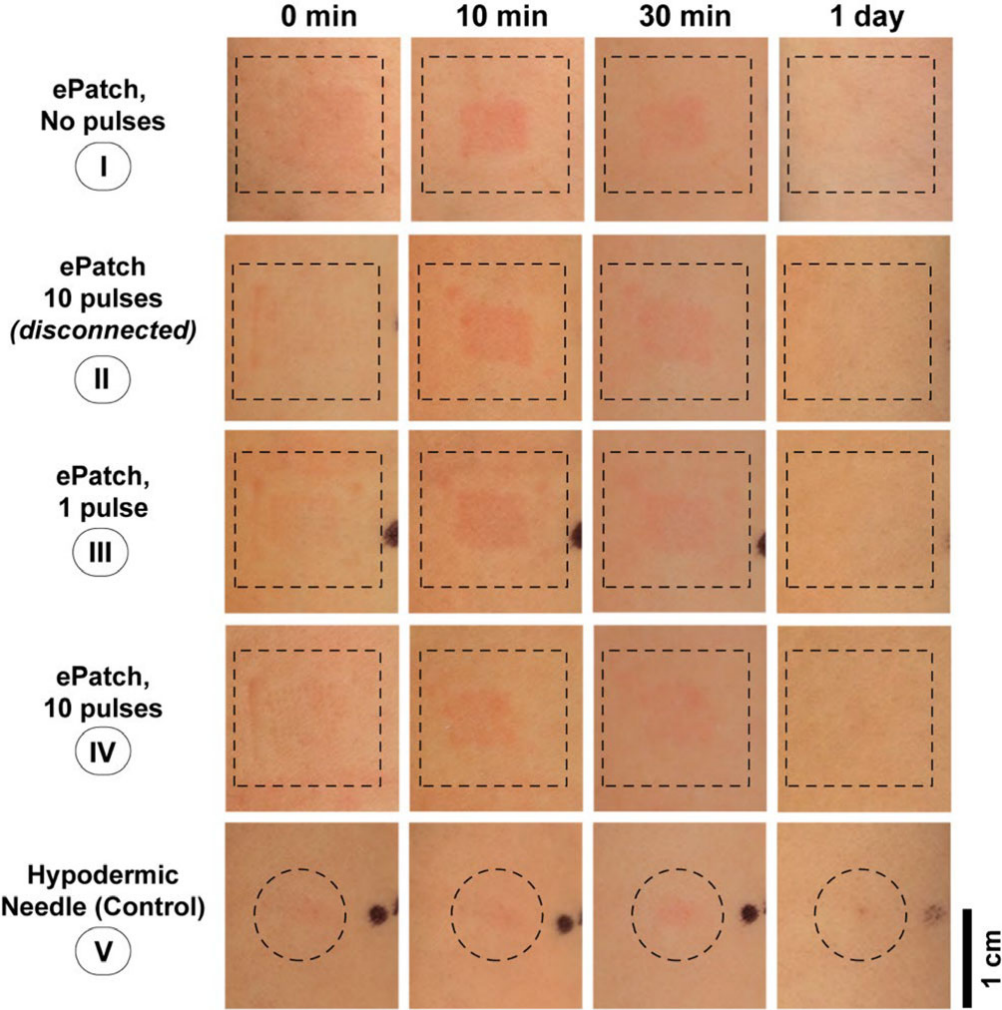
Skin appearance after application of ePatch and hypodermic needle control in human subjects. Photographic images of the forearm skin of a representative study participant are presented at 0 min, 10 min, 30 min or 1 day after skin treatment with ePatch or hypodermic needle insertion. The dashed line identifies sites of ePatch or hypodermic needle treatment. Black ink dots were applied to the skin to identify treatment sites.

To supplement quantitative skin tolerability assessments in Figures 3 and 4, we imaged the skin by digital photography to provide a qualitative assessment of visual appearance at multiple time points (Figure 5). Immediately after treatment, mild skin indentation caused by MEA insertion was evident at sites of ePatch application, and the site of hypodermic needle puncture could be seen too (Figure 5 @0 min). Erythema developed over time, becoming clear (albeit mild) after 10 min generally in an area up to 1 cm^2^ at ePatch application sites (Figure 5 @10 min). Erythema was not commonly observed at hypodermic needle sites, but when present, it was concentrated around the puncture and covered a smaller area.

After 30 min, erythema was still seen, but became less pronounced at sites of ePatch treatment; however, the appearance of erythema remained similar at the hypodermic needle sites (Figure 5 @30 min). The following day, there were no signs of erythema at the ePatch or hypodermic needle administration sites (Figure 5 @1 day).

### 3.5 Tolerability of ePatch in rats

**FIGURE 6:**
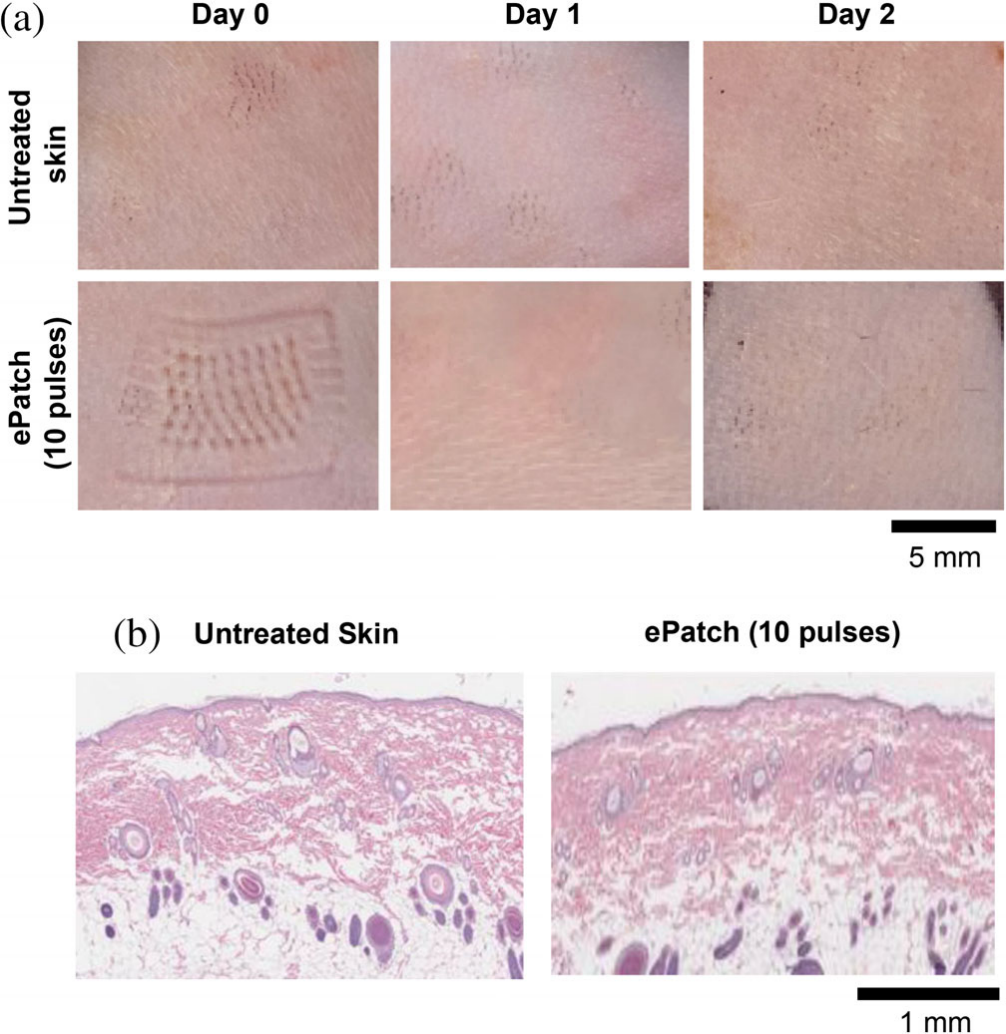
Skin tolerability after application of ePatch in rats. (a) Representative photographic images of skin appearance over time for untreated skin and for skin after treatment by ePatch with 10 pulses. (b) Representative histological cross sections (H&E stained) of untreated skin and of skin 1 day after treatment by ePatch with 10 pulses.

We also applied ePatch to rats in vivo and assessed skin tolerability. Skin indentation at the MEA puncture site was immediately apparent after applying the ePatch with 10 pulses to rats, similar to human subjects (Figure 5), yet more pronounced (Figure 6a). The skin indentation was no longer evident after 1 day, and erythema and induration/swelling were not observed at any time in the rat skin after ePatch application. Histological examination of rat skin 1 day post‐ePatch application showed no notable differences when compared to neighboring untreated skin from the same rat (Figure 6b), indicating that any skin damage caused by ePatch was no longer evident within 1 day of treatment.

## 4 DISCUSSION

Nucleic acid‐based treatments are already making an impact on COVID vaccination, cancer therapies, and treatments of rare genetic disorders, and promise to offer more medical advances in the future.^3^, ^4^, ^6^ A limitation, however, of these therapies relates to their safety, cost, efficacy and delivery; where delivery requires viral vectors for DNA and LNPs for mRNA.^7^, ^9^, ^29^ While electroporation offers a promising alternative delivery method that is well established in the lab with limited current use in the clinic, further impact of electroporation‐mediated delivery is constrained by the large size, cost, and complexity of commercial electroporators, as well as the unpleasant nerve stimulation that they cause.^20^, ^26^

To make electroporation more accessible to patients and providers, we developed the ePatch to be a simple, inexpensive, and handheld electroporator which showed dramatic improvement in DNA vaccination in rodents.^32^ Guided by the hypothesis that localization of the electric field produced by ePatch to the upper layers of skin could minimize pain and sensation to the upper layers of skin associated with electroporation. In this study, we assessed the pain and skin tolerability of ePatch and compared it to the pain and skin tolerability of inserting a hypodermic needle insertion to the skin of human subjects.

We found that ePatch was well‐tolerated in human subjects and the application of ePatch with 1 or 10 pulses administered to the skin caused significantly less pain compared to the insertion of a hypodermic needle in the skin. We also found that the minimal pain associated with ePatch with 1 or 10 pulses of electroporation was not significantly different from ePatch applied to the skin without pulses or with the simulation of 10 pulses using a disconnected MEA. This finding indicates that participants could not tell the difference between MEA insertion in the skin with and without electroporation pulses, suggesting that nerve stimulation from the electric pulses was imperceptible. The higher pain scores with hypodermic needle insertion were likely due to stimulation of sensory nerves in the skin and subcutaneous tissue, whereas ePatch microneedle electrodes allowed for limited penetration depth into the epidermis and superficial dermis of the skin, causing less pain.

To better understand the pain associated with electroporation by ePatch, we normalized ePatch VAS scores to pain scores of the hypodermic needle control (nVAS_HN_) and of ePatch with no pulses (nVAS_NP_). We found that for all ePatch groups, the value for nVAS_HN_ was almost always less than one for all subjects and all types of ePatch, further indicating that ePatch was considered less painful than a hypodermic needle. The value for nVAS_NP_ was roughly at or below a value of one for all other ePatch test groups, further indicating that ePatch with electric pulses was not perceived to cause more pain than ePatch with no electric pulses.

Skin inspection of human subjects showed that the ePatch treatments consistently caused mild erythema that resolved in 1 day, but there was no apparent difference in the size, intensity, or duration of erythema for an ePatch administering electric pulses versus an ePatch administering no pulses. This indicates that the erythema response was due largely to the MEA and not the electroporation pulses, which is consistent with prior literature showing a similar mild, transient erythema after the application of microneedles to the skin in other contexts.^42^ In contrast, the hypodermic needle was less likely to cause erythema but was more likely to cause residual pain and tenderness than the ePatch. One day post‐ePatch application, all erythema resolved and there were no reports of residual pain, tenderness, or swelling. Companion studies in rats also showed no evidence of skin damage based on a clinical exam of the skin surface for 2 days after ePatch treatment or based on histological examination of skin sections 1 day after ePatch treatment.

Our findings show a stark contrast to the outcomes associated with traditional commercial electroporators known to induce muscle twitching and pain due to nerve stimulation.^35^, ^43^, ^44^ Notably, after intramuscular (IM) electroporation, which delivers 3 pulses (each 52 ms long) via a 5‐needle electrode array, subjects reported a mean immediate VAS pain score of 6.28.^35^ In contrast, intradermal (ID) electroporation via a 3‐needle electrode array in a similar way (4 pulses, 52 ms each) resulted in a significantly lower average VAS score of 2.5, which is comparable to the VAS score for 10 pulses delivered via the ePatch (2.36). This suggests that ePatch may offer a less painful alternative to conventional IM electroporation. In a related study of IM electroporation, out of 10 subjects, seven reported transient tenderness at the site of administration, while two experienced possible adverse neurological events such as hypoesthesia and paresthesia.^35^ In contrast, our study only observed mild erythema, resolving within a day, without any reports of hypoesthesia or paresthesia.

Comparatively, a separate study reported an average VAS pain score of 2.5 following ID electroporation using an array of 1 mm long microneedle electrodes delivering pulses ranging from 0.1 to 10 ms.^43^ Although this pain score is comparable to the one observed with the ePatch in our study, subjects experienced residual pain lasting 30–60 min after the conventional ID electroporation, a side effect absent in ePatch users.^35^ This suggests that skin electroporation, particularly with ePatch's shorter electrodes and microsecond‐long biphasic pulses, may be inherently less painful than IM electroporation. Moreover, the ePatch's design may minimize the potential for lasting pain typically associated with the longer, millisecond‐long monophasic pulses utilized by conventional electroporators.^35^, ^44^

Future research should extend the evaluation of ePatch's safety, tolerability, and acceptability using both placebo comparisons and actual therapeutic delivery of DNA, mRNA, or other therapeutic agents. Given the limited size and scope of the current participant pool, future studies would benefit from a larger and more diverse sample population, encompassing a wide range of racial, ethnic, and age demographics. Investigating the effects of ePatch on various skin sites beyond the forearm will provide a more comprehensive understanding of its application.

## 5 CONCLUSION

Electroporation stands as a compelling delivery system for nucleic acids, offering an attractive alternative to traditional viral and LNP methods. However, the widespread implementation of electroporation has been hindered by the complexity, expense, and patient discomfort associated with conventional devices. Our study evaluates the ePatch, a user‐friendly, cost‐effective piezoelectric electroporator that specifically targets the epidermis and superficial dermis to minimize nerve stimulation. In a cohort of 14 healthy volunteers, the ePatch demonstrated a high degree of tolerability, eliciting minimal discomfort even with the administration of up to 10 pulses. Notably, the pain experienced during ePatch application was significantly less than that associated with traditional hypodermic needle insertion. Crucially, there was no discernible difference in pain, tenderness, or swelling when active ePatch electroporation was compared to a placebo ePatch without electric pulse administration. The mild, transient erythema observed in all ePatch recipients resolved within 24 h, further underscoring the mild nature of the device's skin interaction. Our findings indicate that the ePatch's design can effectively mitigate the primary drawbacks of existing electroporation techniques, thereby enhancing patient tolerability while maintaining efficacy. These promising results further support the ePatch as a well‐tolerated electroporation modality for the delivery of nucleic acid‐based therapies. The potential for the ePatch to improve the delivery of genetic medicine is significant, warranting further investigation and optimization.

## AUTHOR CONTRIBUTIONS

**Chao‐Yi Lu:** Data curation; formal analysis; investigation; methodology; software; validation; visualization; writing – original draft. **Pankaj Rohilla:** Data curation; formal analysis; investigation; methodology; software; validation; visualization; writing – original draft. **Eric I. Felner:** Data curation; investigation; methodology; supervision; validation; writing – review and editing. **Gaurav Byagathvalli:** Conceptualization; methodology; validation; writing – review and editing. **Erkan Azizoglu:** Investigation; writing – review and editing. **M. Saad Bhamla:** Conceptualization; funding acquisition; methodology; resources; supervision; validation; writing – review and editing. **Mark Prausnitz:** Conceptualization; funding acquisition; methodology; project administration; resources; supervision; validation; writing – review and editing.

## CONFLICT OF INTEREST STATEMENT

MSB, GB and MRP are inventors of patents and co‐founders of Piezo Therapeutics, which is commercializing ePatch technology. Georgia Institute of Technology manages the associated conflicts of interest through its established procedures. The remaining authors declare no conflicts of interest.

## Supporting information


**Figure S1.** Force measurement during ePatch administration. (a) Temporal force profile during ePatch administration by clinical investigator on porcine skin. ePatch was kept on the skin for the time required to generate 10 electric pulses and this time was kept constant for the human study. (b) Peak force for ePatch application by clinical investigator on porcine skin (*n* = 5).
**Figure S2.** Representative voltage profile recorded during electroporation of skin of a participant via an ePatch. Data were collected by oscilloscope at a sampling rate of 1 GSa/s, and then smoothed to an effective sampling rate of 0.5 GSa/s using Savitzky–Golay filter to remove noise.
**Table S1.** Skin score sheet used to measure skin tolerability.
**Table S2.** Demographics of study participants.

## Data Availability

The data that support the findings of this study are available from the corresponding author upon reasonable request.
