# Supplementary material for: Tolerability of a piezoelectric microneedle electroporator in human subjects
Source: Bioeng Transl Med. 2024 Mar 14;9(4):e10662. doi: 10.1002/btm2.10662 (PMC11256137; doi:10.1002/btm2.10662)
Supplement: Supplementary file 1 — Figure S1. Force measurement during ePatch administration. (a) Temporal force profile during ePatch administration by clinical investigator on porcine skin. ePatch was kept on the skin for the time required to generate 10 electric pulses and this time was kept constant for the human study. (b) Peak force for ePatch application by clinical investigator on porcine skin (n = 5). Figure S2. Representative voltage profile recorded during electroporation of skin of a participant via an ePatch. Data were collected by oscilloscope at a sampling rate of 1 GSa/s, and then smoothed to an effective sampling rate of 0.5 GSa/s using Savitzky–Golay filter to remove noise. Table S1. Skin score sheet used to measure skin tolerability. Table S2. Demographics of study participants. [file BTM2-9-e10662-s001.docx]

**SUPPLEMENTARY INFORMATION**

**TOLERABILITY OF A PIEZOELECTRIC MICRONEEDLE ELECTROPORATOR IN HUMAN SUBJECTS**

Chao-Yi Lu^1,#^, Pankaj Rohilla^2,#^, Eric I. Felner^3^, Gaurav Byagathvalli^2,4^, Erkan Azizoglu^2^, M. Saad Bhamla^2, *^, and Mark R. Prausnitz^1, 2, *^

^1^Wallace H. Coulter Department of Biomedical Engineering AT Georgia Tech and Emory University, Georgia Institute of Technology, Atlanta, GA 30332

^2^School of Chemical and Biomolecular Engineering, Georgia Institute of Technology, Atlanta, GA 30332

^3^Department of Pediatrics, Division of Endocrinology, Emory University School of Medicine, Atlanta, GA 30322, USA

^4^Piezo Therapeutics, Atlanta, GA 30303, USA


*^#^These authors contributed equally.*

**To whom correspondence should be addressed*: [saadb@chbe.gatech.edu](mailto:saadb@chbe.gatech.edu), [prausnitz@gatech.edu](mailto:prausnitz@gatech.edu)

**
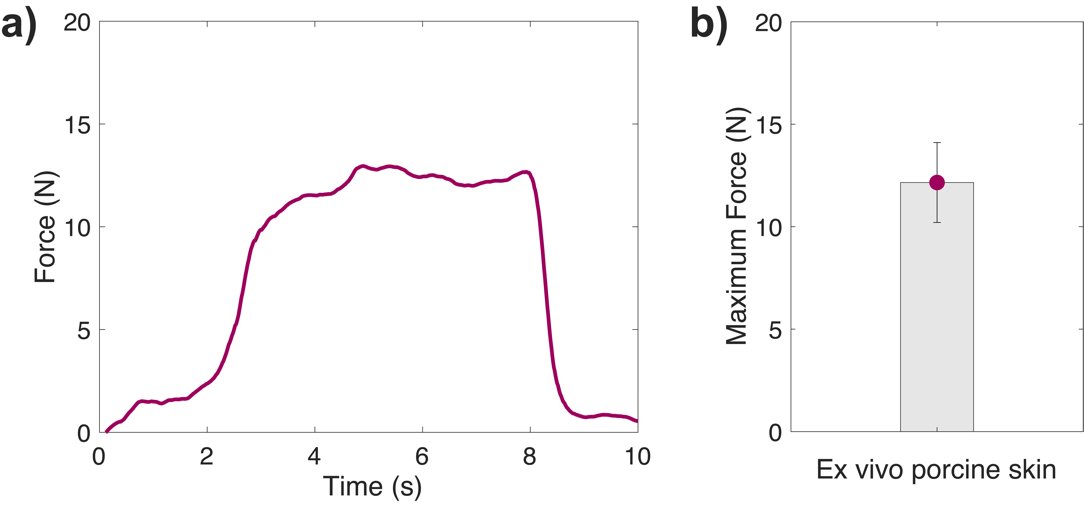
**

**Figure S1. Force measurement during ePatch administration.** (a) Temporal force profile during ePatch administration by clinical investigator on porcine skin. ePatch was kept on the skin for the time required to generate 10 electric pulses and this time was kept constant for the human study. (b) Peak force for ePatch application by clinical investigator on porcine skin (n=5).


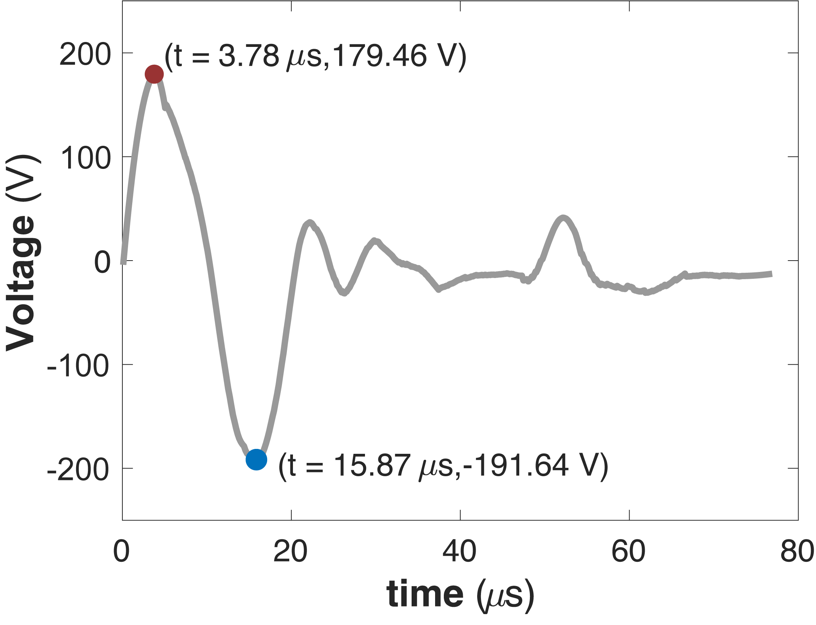


**Figure S2: Representative voltage profile recorded during electroporation of skin of a participant via an ePatch.** Data were collected by oscilloscope at a sampling rate of 1 GSa/s, and then smoothed to an effective sampling rate of 0.5 GSa/s using Savitzky-Golay filter to remove noise.

**Table S1: Skin score sheet used to measure skin tolerability.**

|  |  |  |  | **Grade** |  |  |
| --- | --- | --- | --- | --- | --- | --- |
|  | 0 | 0.5^a^ | 1 | 2 | 3 | 4 |
| **Pain** | No pain | N/A | Mild pain that does not interfere with activity | Repeated use of nonnarcotic pain reliever >24 hrs. or interference with activity | Any use of narcotic pain reliever or prevents daily activity | Emergency room (ER) visit or hospitalization |
| **Tenderness** | No discomfort to touch | N/A | Mild discomfort  to touch | Discomfort with movement | Significant discomfort at rest | ER visit or  hospitalization |
| **Erythema**  **Size^b^** | 0 cm | 0.1-1 cm | 1.1-5 cm | 5.1-10 cm | >10 cm | Necrosis or exfoliative dermatitis |
| **Erythema**  **intensity** | No erythema | N/A | Very slight erythema  (barely perceptible) | Well-defined erythema | Moderate to severe erythema | Severe erythema  (beet redness) |
| **Induration**  **/Swelling** | 0 cm | 0.1-1 cm | 1.1-5 cm and does not  interfere with activity | 5.1-10 cm or interferes with activity | >10 cm or prevents  daily activity | Necrosis |

^a^A score of 0.5 was used for erythema size and swelling only. ^b^Size is characterized as the diameter of the skin area with erythema.

**Table S2: Demographics of study participants**

|  | **Age (years)** |
| --- | --- |
| Mean ± SD | 25.2 ± 2.9 |
| Mode (IQR) | 25 (24-27) |
|  |  |
|  |  |
| **Sex** | **Number of Participants** |
| Male (%) | 6 (~43%) |
| Female (%) | 8 (~57%) |
|  |  |
|  |  |
| **Race** | **Number of Participants** |
| White (%) | 8 (~57%) |
| Black (%) | 1 (~7%) |
| Asian (%) | 5 (~36%) |
|  |  |
|  |  |
| **Ethnicity** | **Number of Participants** |
| Hispanic | 6 (~43%) |
| Non-Hispanic | 8 (~57%) |
